# Safety and Immunogenicity of SARS-CoV-2 mRNA Vaccine Booster Doses in Kidney Transplant Recipients: Results of a 12-mo Follow-up From a Prospective Observational Study

**DOI:** 10.1097/TXD.0000000000001645

**Published:** 2024-05-17

**Authors:** Vojtech Petr, Ivan Zahradka, Istvan Modos, Matej Roder, Martina Fialova, Jana Machkova, Katerina Kabrtova, Petra Hruba, Maria Magicova, Antonij Slavcev, Ilja Striz, Ondrej Viklicky

**Affiliations:** 1 Department of Nephrology, Institute for Clinical and Experimental Medicine, Prague, Czech Republic.; 2 Information Technology Department, Institute for Clinical and Experimental Medicine, Prague, Czech Republic.; 3 Immunogenetics Department, Institute for Clinical and Experimental Medicine, Prague, Czech Republic.; 4 Department of Clinical and Transplant Immunology, Institute for Clinical and Experimental Medicine, Prague, Czech Republic.; 5 Transplant Laboratory, Institute for Clinical and Experimental Medicine, Prague, Czech Republic.

## Abstract

**Background.:**

Booster doses of SARS-CoV-2 mRNA vaccines are commonly used in kidney transplant recipients (KTRs). However, there is uncertainty regarding the waning of vaccination responses and immunological safety in KTRs.

**Methods.:**

A total of 123 KTRs were included in the final analysis of this prospective observational cohort study. The aim was to evaluate the immunogenicity and immunological safety. SARS-CoV-2 antispike IgG antibodies and anti-HLA antibodies were measured at baseline and then at months 3, 6, and 12 after vaccination with the first booster dose (ie, the third vaccine dose). Antibodies against S1 and S2 subunits of SARS-CoV-2 were evaluated using an immunochemiluminescent assay (cutoff 9.5 AU/mL, sensitivity 91.2%, and specificity 90.2%). Anti-HLA antibodies were analyzed using single-antigen bead technology.

**Results.:**

Seroconversion was reached in 65% of KTRs previously nonresponding to 2-dose mRNA vaccination; the overall seroconversion rate 3 mo after the first booster dose was 83%. Vaccination induced a durable humoral response, and the antibody levels were stable during the 12-mo study follow-up. Higher age (exponentiated beta coefficient [e^β^] 0.97; 95% confidence interval [CI], 0.943-0.997) and a full dose of mycophenolate (e^β^ 0.296; 95% CI, 0.089-0.984) were negatively associated with SARS-CoV-2 IgG antibody levels, whereas better graft function (e^β^1.021; 95% CI, 1.005-1.037) was associated positively. There were no systematic signs of anti-HLA antibody development after vaccination. However, during the follow-up, there was a nonsignificant signal of an increase in anti-HLA antibodies in those who developed COVID-19.

**Conclusions.:**

Additional booster doses of SARS-CoV-2 mRNA vaccines induce durable antibody response even in a large subset of previous nonresponders and are not associated with the risk of allosensitization. Furthermore, a signal linking COVID-19 to the development of anti-HLA antibodies was observed, and this should be confirmed and further examined (NCT05483725).

Infections remain one of the most common causes of death in kidney transplant recipients (KTRs), a fact even more highlighted during the COVID-19 pandemic.^1^ Novel SARS-CoV-2 mRNA vaccines have quickly become a cornerstone of COVID-19 prevention in vulnerable populations. However, the initial serological studies showed a suboptimal humoral response to mRNA vaccines among KTRs.^2,3^ Although SARS-CoV-2 mRNA vaccines were shown to be effective in KTRs in real-world settings,^4,5^ an extent of this protection remains unclear. This is even more pressing because the protection provided by 2 doses of mRNA vaccines substantially wanes with time and the emergence of new viral variants.^6^

Interestingly, vaccination in organ transplant recipients has been widely discussed regarding its safety, although no evidence of increased rejection or autoimmunity rates has been shown.^7-11^ Similar concerns have been raised with the advent of new mRNA vaccines.^12^ Because mRNA vaccination is a novel technology whose safety has not yet been comprehensively studied in KTRs, establishing its safety regarding alloreactive adverse events is important. Thus, further research of vaccine responses in KTRs is invaluable because it not only provides insights into unexpected impacts of immunosuppression on infections and immunization responses but can also bring important postmarketing safety data and information about long-term vaccine effectiveness and antibody durability.

There were no safety signals in KTRs who received 3 doses of mRNA vaccines in the short-term follow-up.^7^ Here, we show the 12-mo follow-up of a prospective, observational study aimed at assessing its immunogenicity and immunological safety (NCT05483725, EudraCT No. 2022-000319-30).

## MATERIALS AND METHODS

### Study Design

Here, we report the results of a 12-mo follow-up of a prospective single-center observational cohort study regarding the immunological safety and immunogenicity of a third (ie, the first booster) dose of SARS-CoV-2 mRNA vaccine in KTRs. Immunogenicity was assessed by the dynamic of SARS-CoV-2 antispike IgG antibodies, whereas immunological safety was assessed by anti-HLA antibody measurements.

Subjects considered for inclusion were adult KTRs, who were previously vaccinated with 2 doses of SARS-CoV-2 mRNA vaccines and were scheduled for the administration of a third vaccine dose. Subjects were enrolled between October 4, 2021, and December 3, 2021. All previous SARS-CoV-2 infection records were verified in the official government-run registry (Information System for Infectious Diseases),^13^ into which all positive polymerase chain reaction and antigen test results from laboratories throughout the country were mandatorily reported. Blood samples were collected on the day of the first booster dose administration (day 0 visit, D0 visit) and subsequently 3 mo (month 3 visit, M3), 6 mo (month 6 visit, M6), and 12 mo (month 12 visit, M12) after, respectively. Medians and interquartile ranges as indicators of the spread of sampling days are provided in Table S1 (**SDC,** http://links.lww.com/TXD/A655). Additionally, information about the immunosuppression at the D0 visit (tacrolimus, cyclosporin A, or mechanistic target of rapamycin inhibitor levels, respectively, a daily dose of mycophenolate, and induction immunosuppression) was recorded.

The vaccine applied was BNT162b2 to all participants.

### Study Population

**FIGURE 1.**
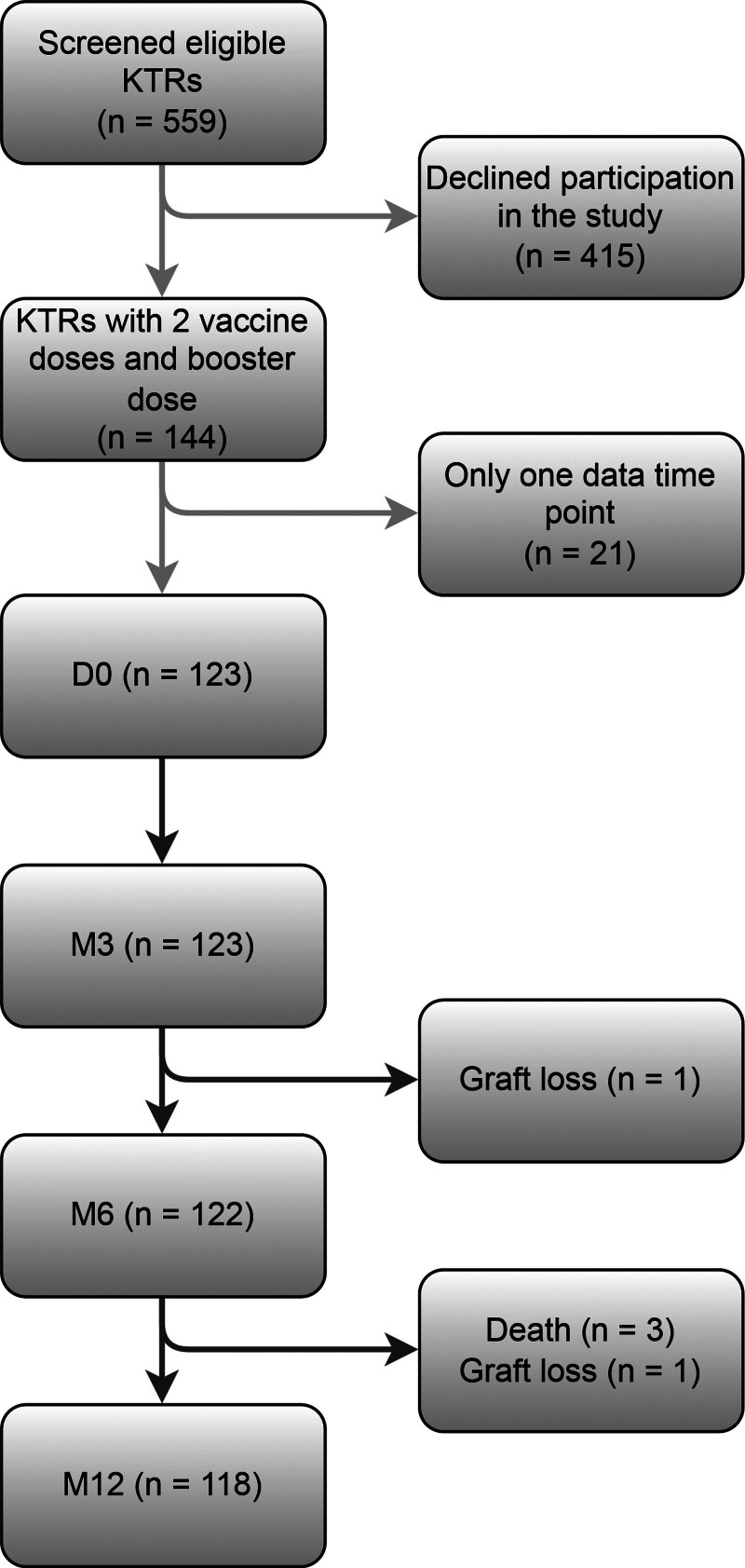
Study flowchart. Five hundred fifty-nine KTRs were considered for inclusion, 415 did not consent to participate, and 144 were enrolled in the study. One hundred twenty-three KTRs were included in the final analysis, because 21 enrolled KTRs attended only the D0 visit. Furthermore, 1 participant experienced graft loss between 3 and 6 mo visit, and 1 participant experienced graft loss, and 3 participants died between 6 and 12 mo visit. KTR, kidney transplant recipient.

In total, 559 KTRs were considered for inclusion; 415 did not consent to participate, and 144 were enrolled in the study. Finally, we included 123 KTRs in the final analysis, because 21 enrolled KTRs attended only the D0 visit. Furthermore, 1 participant experienced graft loss between 3- and 6-mo visits, and 3 participants died, and 1 experienced graft loss between 6- and 12-mo visits (Figure 1). Of the KTRs who died during the follow-up, 1 died at age 79 because of mesenteric ischemia, 1 died at age 72 because of terminal heart failure, and 1 died at age 69 because of multiorgan failure in COVID-19.

Written informed consent and consent to personal data processing were obtained from all participants before their enrollment. The ethical board approved this study under No. G-21-71. This study is approved and overseen by the State Institute for Drug Control, and it is registered with EudraCT number 2022-000319-30 and NCT05483725. The study is in accordance with the 1964 Declaration of Helsinki and its later amendments. The clinical and research activities being reported are consistent with the Principles of the Declaration of Istanbul as outlined in the “Declaration of Istanbul on Organ Trafficking and Transplant Tourism.”

### Assessment of Clinical Adverse Events

Study participants were contacted by phone or e-mail by a physician (M.M.) and were asked specifically about any reactions to the booster vaccination. Participants were contacted within 2 to 4 wk after vaccine administration. Monitored adverse events included but were not limited to anaphylaxis, hypersensitivity, allergic reactions, local symptoms at the injection site (pain, swelling, and redness), fatigue, elevated body temperature (37–37.9 °C or ≥38 °C), chills and shivers, headache, muscle aches, joint aches, nausea, vomiting, diarrhea, insomnia, facial nerve palsy, swelling of or pain in lymph nodes, infection, and death.

### Detection of Anti-HLA Antibodies and Donor-specific Antibodies

Anti-HLA antibodies were analyzed in samples from all study visits (D0, M3, M6, and M12) using the single-antigen bead technology. Screening was performed using the LabScreen Mixed, and in case of a positive result, single antibodies were tested by the LabScreen Single Antigen Luminex technique (both One Lambda Inc). Donor HLA typing from the time of transplantation was used for the assessment of donor-specific antibodies (DSAs). Organ donors were typed for HLA-A, -B, -DR, and -DQ loci (polymerase chain reaction sequence-specific primer [SSP] low-resolution kits, Olerup SSP, and Histo Type SSP, BAG). The presence of DSAs was assessed using HLA fusion version 4.6.0 software (One Lambda Inc). Both the emergence of de novo anti-HLA antibodies and the dynamics of preformed antibodies were evaluated. The laboratory cutoff of 2000 mean fluorescence intensity (MFI) was set as the threshold for HLA-specific antibody positivity because it is the validated threshold by the local immunogenetics laboratory. A significant increase in anti-HLA antibodies or DSAs was defined as an emergence of a de novo antibody finding with MFI of >2000 or a > 50% increase in MFI of a preformed antibody. Furthermore, a sensitivity analysis was performed where the threshold of anti-HLA antibody positivity was lowered to MFI of >500 because this threshold was also previously used in other studies and can be considered conservative.^7,14-16^ Calculated virtual panel-reactive antibodies were computed using the Eurotransplant Reference Laboratory calculator.^17^ KTR is considered sensitized if at least 1 anti-HLA antibody of >500 MFI was present at the D0 visit.

### Anti–SARS-CoV-2 Antibody Detection

SARS-CoV-2 antispike IgG antibodies were tested from samples from all study visits using the LIAISON SARS-CoV-2 S1/S2 IgG chemiluminescence immunoassay (DiaSorin S.p.A., Italy). Seroconversion was defined as antibodies level >9.5 arbitrary units (kAU/mL) with a 91.2% sensitivity (95% confidence interval [CI], 76.3-98.1) and 90.2% specificity (95% CI, 76.9-97.3) determined using MedCalc Statistical Software version 19.1. A detailed description of the SARS-CoV-2 antibody detection method was previously described elsewhere.^2^

### Statistics

The statistics were calculated using R, version 4.3.1. Continuous variables are reported as medians with interquartile ranges, and categorical variables are reported as proportions. The Wilcoxon rank-sum test was used to compare continuous variables, and the chi-squared test or Fisher exact test was used to compare categorical variables. Two-sided testing was performed unless stated otherwise.

The unadjusted anti–SARS-CoV-2 IgG antibody dynamics were compared using the paired Wilcoxon test, and the adjusted dynamics were compared using a linear mixed model, in which the antibody level was a dependent variable. The mixed model considers that each KTRs had at most 4 antibody measurements and these measurements can be correlated; random intercepts modeled the variability of each KTR. Moreover, mixed models do not assume a complete-block design, which is why they allow some measurements to be missing. The time from the third dose to anti–SARS-CoV-2 IgG antibody measurement was modeled using a factor variable (with values D0 (baseline), M3 (3 mo visit), M6 (6 mo visit), and M12 (12 mo visit)). The antibody level was log transformed; therefore, the raw models’ coefficients are additive on the log scale; after exponentiating them, the transformed coefficients have a multiplicative effect on the antibody level. To adjust the model to a possible fourth dose orSARS-CoV-2 infection, we included a factor variable indicating the time since the fourth dose or SARS-CoV-2 infection (both events have separate variables) to antibody measurement; the reference value of these variables represents that a KTR did not get the fourth dose, or KTR was not infected by SARS-CoV-2.

The anti-HLA antibodies were analyzed with a 1-sided paired Wilcoxon signed-rank test (the alternative hypothesis being that the antibody levels are lower at baseline than in the subsequent months).

All tests were performed at the 5% level of significance.

## RESULTS

### Patient Demographic and Baseline Characteristics

A total of 144 of KTRs were vaccinated and enrolled in the study (Figure 1). We excluded 21 participants for having only 1 data time point; thus, the final analysis included 123 KTRs, 73% were male individuals, the median age was 58 y, the estimated glomerular filtration rate at vaccination was 52 mL/min/1.73 m^2^, and 85% of KTRs were maintained with the standard triple combination (tacrolimus, mycophenolate, and corticosteroids). Ninety-two percent of KTRs were given mycophenolate mofetil or mycophenolic acid (MMF/MPA) at a standard dose (500 mg 2× per day of MMF or 360 mg 2× per day of MPA), 16% had reduced MMF/MPA dose, and 8.1% had no MMF/MPA. Fifty-one percent of KTRs were given the fourth mRNA vaccine dose and 32% were infected with SARS-CoV-2 during the follow-up period, respectively. There was 1 case of graft failure and 4 deaths during the follow-up period. An overview of patient demographics and baseline characteristics is shown in Table 1. The most common adverse event was discomfort at the application site (44%); other adverse events were scarce, and summary is shown in Table S2 (**SDC,** http://links.lww.com/TXD/A655).

**TABLE 1.** Characteristics and baseline demographic

Characteristic	(N = 123)
Male sex, n (%)	90 (73%)
Age at third dose, median (IQR)	58 (49–68)
BMI at vaccination, median (IQR)	28.2 (25.2–32.7)
eGFR at vaccination, median (IQR)	52 (36, 67)
Third dose vaccine type: BNT162b2, n (%)	123 (100)
Anti-HLA antibodies, n (%)	41 (33%)
Preexisting DSA, n (%)	11 (8.9%)
Retransplantation, n (%)	13 (11%)
Years between transplantation and booster dose, median (IQR)	7.9 (3.9–13.8)
Transplantation longer than a year before the booster dose, n (%)	122 (99%)
Diabetes mellitus, n (%)	41 (33%)
Maintenance immunosuppression	
Tacrolimus, n (%)	104 (85%)
Cyclosporine A, n (%)	9 (7.3%)
MMF/MPA	113 (92%)
Standard dose of MMF/MPA	93 (76%)
Reduced dose of MMF/MPA	20 (16%)
No MMF/MPA	10 (8.1%)
mTORi	10 (8.1%)
Corticosteroids	111 (90%)
Fourth dose administered during the follow-up period, n (%)	63 (51%)
COVID-19 infection during the follow-up period, n (%)	39 (32%)

BMI, body mass index; DSA, donor-specific antibody; eGFR, estimated glomerular filtration rate; IQR, interquartile range; MMF, mycophenolate mofetil; MPA, mycophenolic acid; mTORi, inhibitor of mechanistic target of rapamycin.

### Anti–SARS-CoV-2 IgG Antibody Dynamics

Anti–SARS-CoV-2 IgG antibody level and anti-HLA antibody measurements were available in 100% (n = 123) at vaccination, 99.2% (n = 122) at 3 mo, 93.5% (n = 115) at 6 mo, and 91.1% (n = 112) at 12 mo.

**FIGURE 2.**
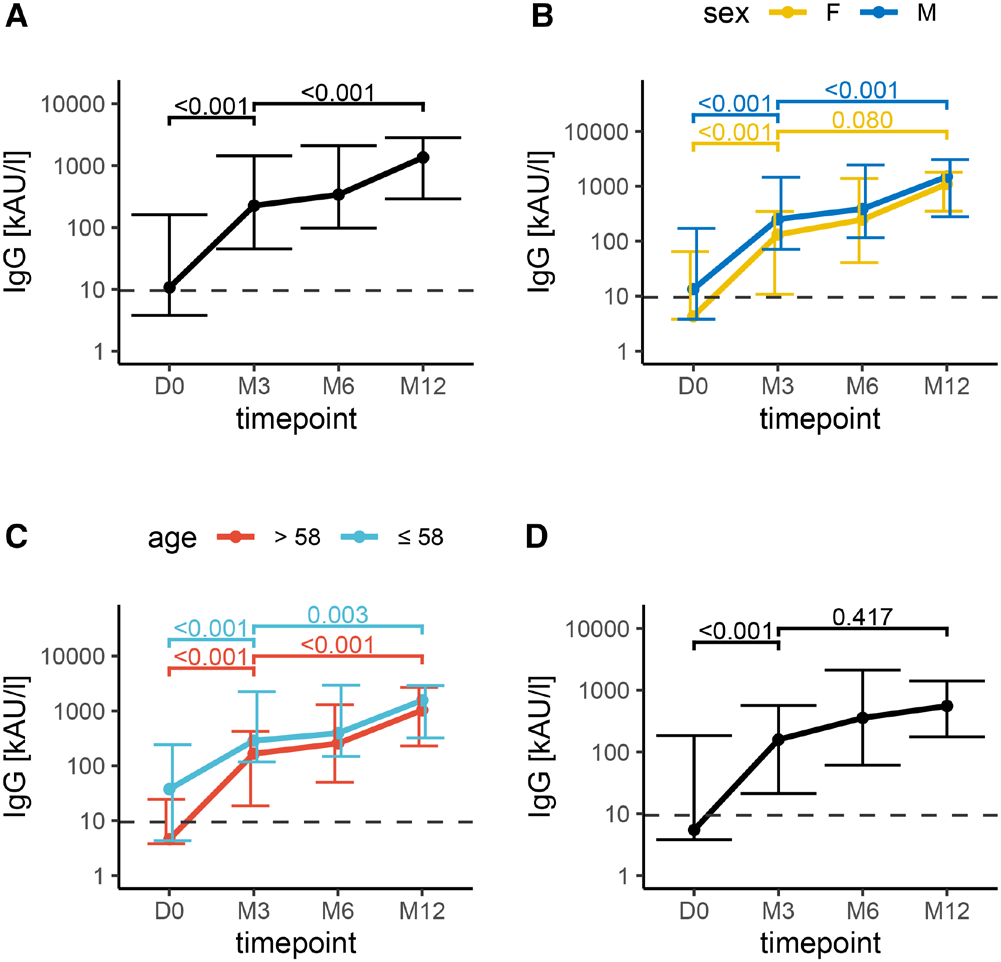
Anti–SARS-CoV-2 IgG levels development in all 4 time points. The y-axis shows IgG antibody levels, log transformed. X-axes show time points: D0: baseline, M3: 3 mo, M6: 6 mo, and M12: 12 mo—(A) all data, (B) antibody levels based on sex, (C) antibody levels in 2 groups: older than median and younger than median age, and (D) antibody levels after exclusion of KTRs who were either administered fourth dose during the follow-up or were infected with SARSCoV-2. The statistical significance was computed using the paired Wilcoxon test. KTR, kidney transplant recipient.

First, we plotted the antibody level development into line graphs. The median antibody level trajectories are depicted in Figure 2. Total SARS-CoV-2 IgG antibody levels first increased after vaccination with the third dose (*P* < 0.001; paired Wilcoxon test; Figure 2A) and then were stable with a slightly increasing trend during the 12-mo follow-up (*P* < 0.001; Figure 2A). Similar trajectories were observed when KTRs were divided into groups according to sex (Figure 2B) or according to median age (>58 and ≤58; Figure 2C), although for female individuals, the difference in SARS-CoV-2 IgG antibody levels between the 3 and 12 mo from the third dose was not statistically significant. Significant increases in IgG antibody levels in 3 mo after third dose were also seen when looking in isolation at KTRs who did not experience any additional event that would lead to stimulation of SARS-CoV-2 IgG antibody production (ie, after the exclusion of KTRs who were infected with COVID-19 or vaccinated with an additional booster dose [ie, the fourth dose]; *P* < 0.001; Figure 2D).

Median anti–SARS-CoV-2 antibody levels are shown in Table 2. At baseline before the application of the first booster dose, seroconversion was observed only in 51% of participants, whereas 3 mo after the first booster dose, the proportion rose to 83%, and 1 y after the booster dose, seroconversion was achieved in 96% of participants. When we excluded those who were either administered fourth dose or were infected with COVID-19 during the follow-up, the seroconversion was detectable in 80% of KTRs at 3 mo and 93% of KTRs at 12 mo.

**TABLE 2.** Anti–SARS-CoV-2 antibody levels and seroconversion rates

Characteristic	Baseline (N = 123)	3 mo (N = 122)	6 mo (N = 114)	12 mo (N = 115)
Seroconversion, n (%)	63 (51%)	101 (83%)	99 (87%)	110 (96%)
Absolute levels of anti–SARS-CoV-2 IgG, IQR (median)	11 (4–160)	226 (45–1 444)	341 (95–2 110)	1360 (289–2 840)
Log_10_ transformed anti–SARS-CoV-2 IgG, IQR (median)	1.03 (0.58–2.20)	2.35 (1.65–3.16)	2.53 (1.99–3.32)	3.13 (2.46–3.45)

IQR, interquartile range; Log_10_, decadic logarithm.

### Predictors of Anti–SARS-CoV-2 IgG Antibody Responses

Next, we modeled the antibody levels with a linear mixed model; the results of a multivariable regression model are shown in Table 3. Better graft function (exponentiated beta coefficient (e^β^)1.021; 95% CI, 1.005-1.037) was independently associated with higher anti–SARS-CoV-2 IgG antibody levels. In accordance with the raw data development (Figure 2), further time points were associated with higher anti–SARS-CoV-2 IgG levels. Second booster dose and COVID-19 infection up to 3 mo before study enrollment were associated with higher anti–SARS-CoV-2 IgG antibody levels. Conversely, higher age (e^β^0.97; 95% CI, 0.943-0.997) and a standard dose of mycophenolate conferred a negative effect (e^β^ 0.296; 95% CI, 0.089-0.984).

**TABLE 3.** Multivariable linear mixed model with anti–SARS-CoV-2 IgG level as the dependent variable

Parameter	e^β^	95% CI	*P*
Intercept	122.319	7.020, 2131.5)	**0.001**
Male sex	1.649	0.774, 3.513)	0.193
Age at third dose, y	0.970	0.943, 0.997)	**0.028**
Body mass index, kg/m^2^	0.988	0.924, 1.056)	0.723
eGFR at vaccination, mL/min/1.73 m^2^	1.021	1.005, 1.037)	**0.008**
Baseline anti-HLA antibodies positivity	1.707	0.761, 3.829)	0.193
Time point			
3 mo	6.246	4.637, 8.412)	**<0.001**
6 mo	8.060	5.881, 11.047)	**<0.001**
12 mo	10.750	(7.234, 15.975)	**<0.001**
Reduced dose of MMF/MPA	0.366	(0.091, 1.478)	0.156
Standard dose of MMF/MPA	0.296	(0.089, 0.984)	**0.047**
Second booster dose (0–2 mo)	2.089	(0.734, 5.950)	0.167
Second booster dose (2–4 mo)	2.696	(1.585, 4.585)	**<0.001**
COVID-19 infection (0–3 mo)	8.597	(4.839, 15.271)	**<0.001**
COVID-19 infection (3–6 mo)	5.665	(2.659, 12.070)	**<0.001**
COVID-19 infection (6–9 mo)	6.609	(3.109, 14.052)	**<0.001**
COVID-19 infection (9–12 mo)	5.950	(2.500, 14.157)	**<0.001**
COVID-19 infection (12–15 mo)	3.581	(1.127, 11.382)	**0.031**
COVID-19 infection (>15 mo)	2.470	(0.797, 7.652)	0.117

e^β^ is used because the anti–SARS-CoV-2 IgG levels were log transformed. The interpretation is as follows: the level of IgG is multiplied by the e^β^ value if the independent variables change by 1.

CI, confidence interval; eGFR, estimated glomerular filtration rate; MMF/MPA, mycophenolate mofetil/mycophenolic acid.

### Anti-HLA Antibodies Analysis

**FIGURE 3.**
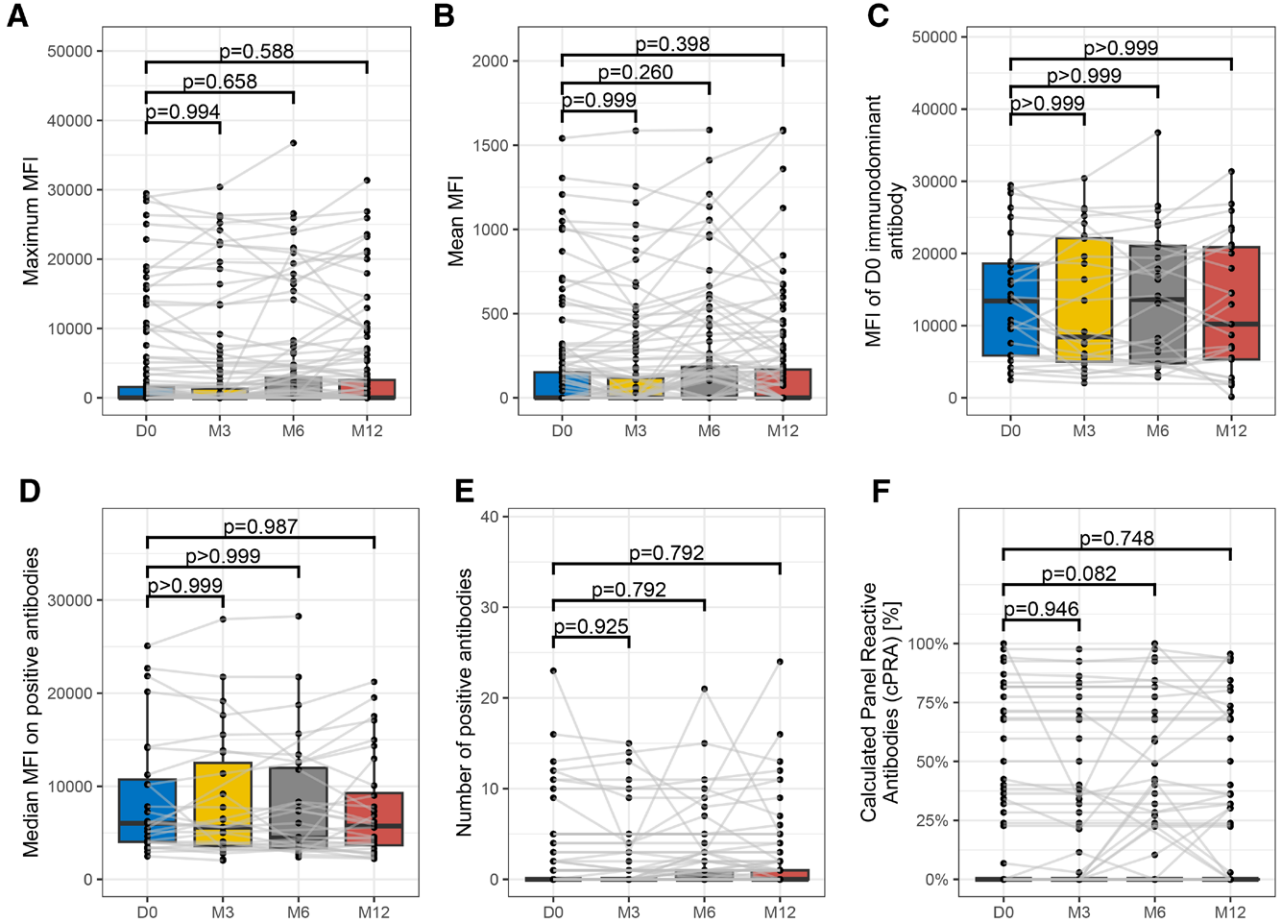
Dynamics of anti-HLA antibody in all 4 time points. X-axes show time points: D0: baseline, M3: 3 mo, M6: 6 mo, and M12: 12 mo. The *P* values show the 1-sided Wilcoxon test comparing baseline and 1 of 3 time points. The statistical tests are in Table S3 (**SDC,** http://links.lww.com/TXD/A655)—(A) peak MFI, (B) mean MFI, (C) MFI of immunodominant anti-HLA antibody at baseline, (D) median MFI of all positive antibodies, (E) counts of positive anti-HLA antibodies, and (F) cPRAs. cPRA, calculated panel-reactive antibody; MFI, mean fluorescence intensity.

Dynamics of both anti-HLA antibodies and DSA were evaluated. We found no significant change in any anti-HLA antibody profiles within the first year postvaccination. Peak MFI, mean MFI, MFI of an immunodominant antibody, and median of all anti-HLA antibodies MFI did not significantly increase during any of the time points compared with the baseline (Figure 3A–D). The number of patients with at least 1 positive anti-HLA antibody (MFI >2000) did not significantly increase during the follow-up period (baseline 78%, 3 mo 78%, 6 mo 72%, and 12 mo 72% of included KTRs had no detectable anti-HLA antibodies; Figure 3E). In these KTRs, the number of targeted HLA antigens also did not change significantly (Figure 3E). Calculated virtual panel-reactive antibodies in all time points did not significantly increase compared with the baseline (Figure 3F). The detailed statistical analysis of anti-HLA antibodies at the 3 time points (3, 6, and 12 mo) compared with baseline is shown in Table S3 (**SDC,** http://links.lww.com/TXD/A655). Furthermore, a sensitivity analysis of anti-HLA antibody dynamics with the use of an alternative threshold to define anti-HLA antibody positivity (MFI >500) was used. This sensitivity analysis confirmed the findings of the main analysis (**Figure S1, SDC,** http://links.lww.com/TXD/A655).

In a sensitivity analysis, we have also modeled the IgG antibody levels with another linear mixed model. This model is the same as the main model shown in Table 3 but uses the alternative anti-HLA antibody positivity threshold (MFI >500 instead of >2000). In this analysis, anti-HLA antibody positivity at baseline was independently associated with higher anti–SARS-CoV-2 IgG antibody levels (**Table S4, SDC,** http://links.lww.com/TXD/A655).

### DSA Analysis

**FIGURE 4.**
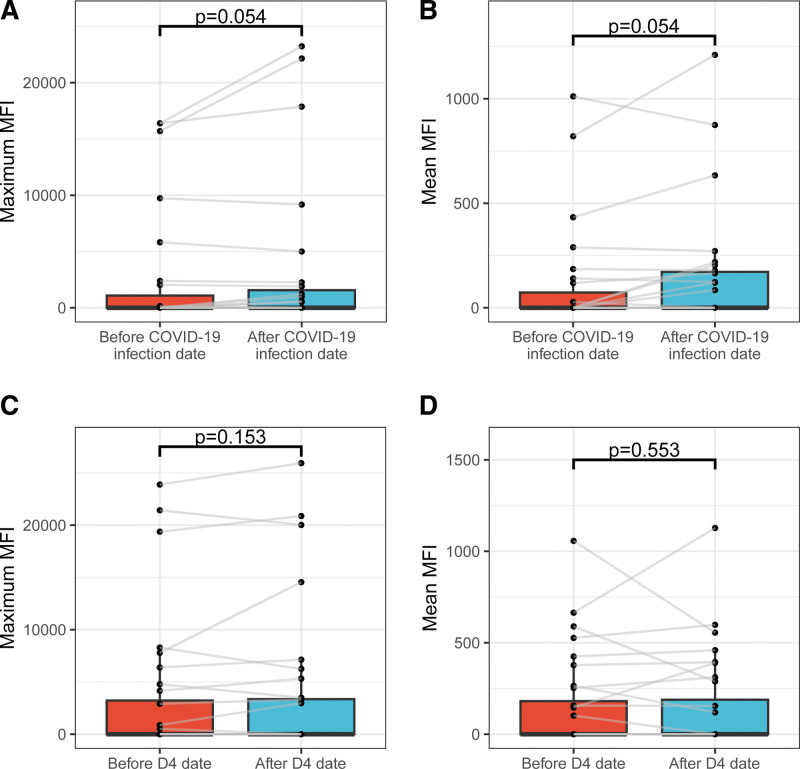
Anti-HLA antibody development in COVID-19-infected subgroup (n = 27) and subgroup administered fourth dose during follow-up (n = 32). The *P* values show the 1-sided Wilcoxon test comparing before and after events (COVID-19 or fourth dose)—(A) maximum MFI before and after COVID-19 infection, (B) mean MFI before and after COVID-19 infection, (C) maximum MFI before and after fourth dose, (D) mean MFI before and after fourth dose. MFI, mean fluorescence intensity.

Of 123 tested KTRs, 10 (8.1%) were found to have at least 1 DSA present at the baseline. Within the year after the booster dose, DSA became negative in 2 participants. In 1 participant, we detected an emergence of de novo DSA of low intensity (2400 MFI); in 1 participant, we detected a significant increase of preformed DSA (9500 MFI to 23 600 MFI). A detailed summary of DSA analysis is shown in Table 4. Interestingly, we also found that anti-HLA antibodies tended to increase in those who were infected with COVID-19, whereas we found no such signal in those who were vaccinated with second booster dose (ie, fourth dose; Figure 4).

**TABLE 4.** Donor-specific antibody MFI during the study follow-up

Patient No.	Targeted HLA	Baseline	3 mo	6 mo	12 mo
1	DQ2	26 400	26 400	24 400	31 400
2	DR53	3700	ND	4500	ND
	DR9	4800	ND	5500	2600
	DR7	3200	ND	ND	ND
3	DP1	2516	2122	**5000**	2500
	DR52	ND	ND	**2200**	ND
4	DQ5	17 000	20 000	19 400	20 900
5	DR13	4700	4800	6000	NA
6	DR53	2500	2200	NA	ND
7	DQ7	25 000	24 200	16 400	23 200
8	B61	1500	ND	ND	ND
9	DQ4	9500	NA	NA	**23 600**
10	DQ2	28 400	26 300	26 600	NA
11	DQ2	23 000	22 100	21 400	20 000
	DQ5	2400	ND	ND	ND
12	DR51	ND	ND	NA	**2400**

MFI, mean fluorescence intensity; NA, not available; ND, not detected.

## DISCUSSION

Vaccination is an essential tool for the prevention of infectious diseases and their complications.^18^ In this prospective, observational study, we aimed to assess the safety and immunogenicity of the first booster dose of the SARS-CoV-2 mRNA vaccine in KTRs. We found that the booster dose was associated with additional seroconversion in almost two-thirds of previously negative KTRs and that a durable IgG antibody response was induced. Furthermore, after the booster dose, we found no clear signs of immune-related adverse events, such as an increase in anti-HLA and DSAs.

There are concerns that protection induced by COVID-19 vaccines will wane over time, especially in patients using immunosuppression.^6,19,20^ In fact, the standard 2-dose vaccination scheme induced an initial peak followed by a significant decline as soon as 12 wk after vaccination, even in healthy individuals.^19,21^ Surprisingly, we found no evidence of antibody level decline after the third mRNA vaccine dose within 12 mo of follow-up. Following the initial increase, the antibody levels further increased, possibly because of factors stimulating antibody production such as booster doses or COVID-19 infections. However, the antibody levels were stable even when these events were excluded. Thus, the humoral response induced by the booster dose was more durable than expected.

However, the vaccination is inevitably associated with an induction of a systematic immune response that might result in rare immune-mediated complications.^8,12,22^ Concerns about vaccination-induced graft rejection have previously been raised among solid organ transplant recipients.^9^ This issue has been addressed several times in various vaccines in the past, and no conclusive evidence of an increased risk of rejection or graft loss has been found.^10,11,23–28^ However, the mRNA vaccine technology is novel and should repeated booster doses become routine, it is even more important to carefully evaluate their safety in the transplanted population. Antibody-mediated graft injury usually results from the presence of anti-HLA antibodies.^29^ The potential induction of anti-HLA antibodies because of vaccination is therefore of significant concern. However, we found no compelling evidence of any increase in anti-HLA antibodies in KTRs after a third vaccine dose. It is likely that the slight changes observed during the follow-up are most likely because of natural fluctuations rather than because of a sensitization linked to SARS-CoV-2 mRNA vaccination. Similarly, we found that fourth dose was not associated with an increase in anti-HLA antibodies, whereas there was a trend of increase of anti-HLA antibodies after COVID-19 infection itself (albeit statistically nonsignificant because of a low number of patients). The mechanisms by which COVID-19 might lead to a generation of anti-HLA antibodies should be confirmed and further studied.

It has been previously suggested that the mechanisms underlying the generation of antibodies against SARS-CoV-2 after vaccination might be similar to those causing the generation of anti-HLA antibodies.^30^ Interestingly, we found an association between higher anti–SARS-CoV-2 levels and baseline presence of anti-HLA antibodies with MFI >500 before booster dose administration.The finding of higher SARS-CoV-2 antibody levels in HLA-sensitized KTRs was observable despite the fact that all HLA-sensitized KTRs except 1 were treated with mycophenolate, which is known to severely impair SARS-CoV-2 antibody production.^2^

The strengths of our study are its prospective design, the length of follow-up, and the longitudinal nature of the assessment, which is one of the main additions of the study. An absence of an unvaccinated control group is the limitation. However, because KTRs are at high risk of a serious course of COVID-19 and even death, it is not ethical to withdraw recommended vaccination from them.^31^ Our center achieved high vaccination rates, and most KTRs who did not receive a booster dose during the study period had either an underlying medical condition or a recent COVID-19 infection. Using these KTRs to form a control group would likely introduce significant bias. Furthermore, another limitation is that we did not measure neutralizing antibodies nor antinucleocapsid antibodies, which might allow identifying patients with previous asymptomatic COVID-19 infections.

In conclusion, this prospective study with a 12-mo follow-up shows that the administration of additional booster doses of SARS-CoV-2 mRNA vaccines in KTRs resulted in an additional SARS-CoV-2 antibody response in a large proportion of previous nonresponders, induced durable antibody response, and seems to be overall safe including the absence allosensitization risk. The results of our study support the administration of booster doses of mRNA vaccines in this vulnerable population.

## Supplementary Material



## References

[1] Ying T Shi B Kelly PJ. Death after kidney transplantation: an analysis by era and time post-transplant. J Am Soc Nephrol. 2020;31:2887–2899. 32908001 10.1681/ASN.2020050566 PMC7790214

[2] Magicova M Zahradka I Fialova M. Determinants of immune response to anti–SARS-CoV-2 mRNA vaccines in kidney transplant recipients: a prospective cohort study. Transplantation. 2022;106:842–852. 34999659 10.1097/TP.0000000000004044 PMC8942601

[3] Sattler A Schrezenmeier E Weber UA. Impaired humoral and cellular immunity after SARS-CoV-2 BNT162b2 (tozinameran) prime-boost vaccination in kidney transplant recipients. J Clin Invest. 2021;131:e150175. 34101623 10.1172/JCI150175 PMC8279581

[4] Zahradka I Petr V Modos I. Association between SARS-CoV-2 messenger RNA vaccines and lower infection rates in kidney transplant recipients: a registry-based report. Ann Intern Med. 2022;175:961–968. 35500256 10.7326/M21-2973 PMC9083501

[5] Aslam S Adler E Mekeel K. Clinical effectiveness of COVID‐19 vaccination in solid organ transplant recipients. Transpl Infect Dis. 2021;23:e13705. 34324256 10.1111/tid.13705 PMC8420394

[6] Levin EG Lustig Y Cohen C. Waning immune humoral response to BNT162b2 Covid-19 vaccine over 6 months. N Engl J Med. 2021;385:e84. 34614326 10.1056/NEJMoa2114583 PMC8522797

[7] Petr V Zahradka I Modos I. First booster of SARS-COV-2 mRNA vaccine is not associated with alloimmunization and subclinical injury of kidney allograft. Transplantation. 2022;107:e62–e64. 36314999 10.1097/TP.0000000000004421

[8] Dudley MZ Halsey NA Omer SB. The state of vaccine safety science: systematic reviews of the evidence. Lancet Infect Dis. 2020;20:e80–e89. 32278359 10.1016/S1473-3099(20)30130-4

[9] Locke JE Zachary AA Warren DS. Proinflammatory events are associated with significant increases in breadth and strength of HLA-specific antibody. Am J Transplant. 2009;9:2136–2139. 19663896 10.1111/j.1600-6143.2009.02764.x

[10] Dendle C Stuart RL Polkinghorne KR. Seroresponses and safety of 13-valent pneumococcal conjugate vaccination in kidney transplant recipients. Transpl Infect Dis. 2018;20:e12866. 29512234 10.1111/tid.12866

[11] Mulley WR Dendle C Ling JEH. Does vaccination in solid-organ transplant recipients result in adverse immunologic sequelae? A systematic review and meta-analysis. J Heart Lung Transplant. 2018;37:844–852. 29609844 10.1016/j.healun.2018.03.001

[12] Goldman M. The safety of anti-SARS-CoV-2 vaccines: vigilance is still required. J Clin Med. 2022;11:1248. 35268339 10.3390/jcm11051248 PMC8910899

[13] Komenda M Bulhart V Karolyi M. Complex reporting of the COVID-19 epidemic in the Czech Republic: use of an interactive web-based app in practice. J Med Internet Res. 2020;22:e19367. 32412422 10.2196/19367 PMC7254961

[14] Süsal C Wettstein D Döhler B; Collaborative Transplant Study Report. Association of kidney graft loss with De Novo produced donor-specific and non-donor-specific HLA antibodies detected by single antigen testing. Transplantation. 2015;99:1976–1980. 25769065 10.1097/TP.0000000000000672

[15] Al Jurdi A Gassen RB Borges TJ. Non-invasive monitoring for rejection in kidney transplant recipients after SARS-CoV-2 mRNA vaccination. Front Immunol. 2022;13:838985. 35281011 10.3389/fimmu.2022.838985 PMC8913529

[16] Reed EF Rao P Zhang Z. Comprehensive assessment and standardization of solid phase multiplex-bead arrays for the detection of antibodies to HLA: CTOT HLA antibody standardization. Am J Transplant. 2013;13:1859–1870. 23763485 10.1111/ajt.12287 PMC3967448

[17] Eurotransplant. Virtual PRA calculator. Available at https://www.etrl.org/vPRA.aspx. Accessed September 25, 2023.

[18] Rodrigues CMC Plotkin SA. Impact of vaccines; health, economic and social perspectives. Front Microbiol. 2020;11:1526. 32760367 10.3389/fmicb.2020.01526 PMC7371956

[19] Pegu A O’Connell SE Schmidt SD; mRNA-1273 Study Group. Durability of mRNA-1273 vaccine–induced antibodies against SARS-CoV-2 variants. Science. 2021;373:1372–1377. 34385356 10.1126/science.abj4176 PMC8691522

[20] Zhuang C Liu X Chen Q. Protection duration of COVID-19 vaccines: waning effectiveness and future perspective. Front Microbiol. 2022;13:828806. 35273584 10.3389/fmicb.2022.828806 PMC8902038

[21] Naaber P Tserel L Kangro K. Dynamics of antibody response to BNT162b2 vaccine after six months: a longitudinal prospective study. Lancet Reg Health Eur. 2021;10:100208. 34514454 10.1016/j.lanepe.2021.100208 PMC8418937

[22] Greinacher A Thiele T Warkentin TE. Thrombotic thrombocytopenia after ChAdOx1 nCov-19 vaccination. N Engl J Med. 2021;384:2092–2101. 33835769 10.1056/NEJMoa2104840 PMC8095372

[23] Blumberg EA Brozena SC Groover JE. Immunogenicity of pneumococcal vaccine (PV) in heart transplant recipients (HTR). Clin Infect Dis. 1997;25:464–464. 10.1086/318482 11170924

[24] Katerinis I Hadaya K Duquesnoy R. De Novo anti-HLA antibody after pandemic H1N1 and seasonal influenza immunization in kidney transplant recipients: H1N1 immunization and anti-HLA antibody. Am J Transplant. 2011;11:1727–1733. 21672157 10.1111/j.1600-6143.2011.03604.x

[25] Brakemeier S Schweiger B Lachmann N. Immune response to an adjuvanted influenza A H1N1 vaccine (Pandemrix(R)) in renal transplant recipients. Nephrol Dial Transplant. 2012;27:423–428. 21613386 10.1093/ndt/gfr278

[26] Cordero E Manuel O. Influenza vaccination in solid-organ transplant recipients. Curr Opin Organ Transplant. 2012;17:601–608. 23042206 10.1097/MOT.0b013e3283592622

[27] Hirzel C Kumar D. Influenza vaccine strategies for solid organ transplant recipients. Curr Opin Infect Dis. 2018;31:309–315. 29771697 10.1097/QCO.0000000000000461

[28] Candon S Thervet E Lebon P. Humoral and cellular immune responses after influenza vaccination in kidney transplant recipients. Am J Transplant. 2009;9:2346–2354. 19656126 10.1111/j.1600-6143.2009.02787.x

[29] Schinstock CA Mannon RB Budde K. Recommended treatment for antibody-mediated rejection after kidney transplantation: the 2019 Expert Consensus from the Transplantion Society Working Group. Transplantation. 2020;104:911–922. 31895348 10.1097/TP.0000000000003095 PMC7176344

[30] Caillard S Thaunat O. COVID-19 vaccination in kidney transplant recipients. Nat Rev Nephrol. 2021;17:785–787. 34580488 10.1038/s41581-021-00491-7 PMC8475856

[31] Zahradka I Petr V Jakubov K. Early referring saved lives in kidney transplant recipients with COVID-19: a beneficial role of telemedicine. Front Med. 2023;10:1252822. 10.3389/fmed.2023.1252822 PMC10546052 37795416

