# Supplementary material for: Safety and Immunogenicity of SARS-CoV-2 mRNA Vaccine Booster Doses in Kidney Transplant Recipients: Results of a 12-mo Follow-up From a Prospective Observational Study
Source: Transplant Direct. 2024 May 17;10(6):e1645. doi: 10.1097/TXD.0000000000001645 (PMC11104726; doi:10.1097/TXD.0000000000001645)
Supplement: Supplementary file 1 [file txd-10-e1645-s001.pdf]

Supplementary Material

Table of Contents **Page #**

**Table S1. Days removed from planned visit date**	**2**
**Table S2. Detailed list of adverse events.**	**3**
**Table S3A – S3F. Statistical analysis of anti-HLA antibodies development**	**4**
**Table S4. Multivariable model of anti-SARS-CoV-2 antibodies with lower threshold for anti-HLA antibody positivity**	**5**
**Figure S1. A sensitivity analysis of anti-HLA antibody dynamics during the follow-up when an alternative cut-off (MFI > 500) is used to define antibody positivity**	**6**

Table S1. Days removed from planned visit date

Timepoint	Median (days)	IQR (days)
3 months visit	-1	-1, -1
6 months visit	-2	-8, 0
12 months visit	-4	-17.5, 3

Table S2. Detailed list of adverse events.

Adverse event	No.	Proportion
Symptoms at application site	48	44.4%
Fatigue	15	13.9%
Headache	4	3.7%
Chills and shivers	2	1.9%
Increased body temperature	2	1.9%
Diarrhea	1	0.9%
Nausea and vomiting	1	0.9%
Arthralgias or back pain	1	0.9%

Table S3. Statistical analysis of anti-HLA antibodies in all timepoints

Table S3A. Peak MFI

Baseline (median (IQR)	Timepoint (median (IQR)	estimate	*p-value*
D0 (0, (0 – 1571.3)	M3 (0, (0 – 1324.2)	414.2	0.994
D0 (0, (0 – 1571.3)	M6 (0, (0 – 2910.3)	-167.6	0.658
D0 (0, (0 – 1571.3)	M12 (0, (0 – 2588.5)	-401.9	0.588

Table S3B. Mean MFI

Baseline (median (IQR)	Timepoint (median (IQR)	estimate	*p-value*
D0 (0, (0 – 151.9)	M3 (0, (0 – 118.7)	45.3	0.999
D0 (0, (0 – 151.9)	M6 (0, (0 – 185.5)	-30.3	0.260
D0 (0, (0 – 151.9)	M12 (0, (0 – 168.4)	-18.1	0.398

Table S3C. Immunodominant antibody MFI

Baseline (median (IQR)	Timepoint (median (IQR)	estimate	*p-value*
D0 (13429.5, (5862.3 – 18615.6)	M3 (8438.7, (5015.7 – 22126.2)	579.2	>0.999
D0 (13429.5, (5862.3 – 18615.6)	M6 (13628.9, (4781.5– 21038.9)	880.2	>0.999
D0 (13429.5, (5862.3 – 18615.6)	M12 (10206.2, (5318.9 – 20873.7)	71.7	>0.999

Table S3D. All antibodies MFI

Baseline (median (IQR)	Timepoint (median (IQR)	estimate	*p-value*
D0 (6020.1, (4031.4 – 10731.4)	M3 (5611, (3582.6 – 12511.1)	-0.86	>0.999
D0 (6020.1, (4031.4 – 10731.4)	M6 (4458.1, (3353.6 – 11973.3)	94.4	>0.999
D0 (6020.1, (4031.4 – 10731.4)	M12 (5727.7, (3654.4 – 9273.3)	-193.5	0.987

Table S3E. Number of positive antibodies

Baseline (median (IQR)	Timepoint (median (IQR)	estimate	*p-value*
D0 (0, (0 – 0)	M3 (0, (0 – 0)	1	0.925
D0 (0, (0 – 0)	M6 (0, (0 – 1)	-0.9	0.792
D0 (0, (0 – 0)	M12 (0, (0 – 1)	-0.00003	0.792

Table S3F. Calculated panel reactive antibodies (cPRA)

Baseline (median (IQR)	Timepoint (median (IQR)	estimate	*p-value*
D0 (0, (0 – 0.7)	M3 (0, (0 – 0.5)	11.6	>0.999
D0 (0, (0 – 0.7)	M6 (0, (0 – 0.7)	-9.5	0.348
D0 (0, (0 – 0.7)	M12 (0, (0 – 0.6)	3.6	>0.999

Note: All p-values are calculated with one-sided Wilcoxon's tests. Estimate is a pseudomedian from D0 and M3, M6 or M12 values. Positive estimate means that D0 value is higher than M3, M6 or M12 value.

Table S4. Multivariable model of anti-SARS-CoV-2 antibody levels with lower threshold for anti-HLA antibody positivity

Parameter		95% CI	p-value
**Intercept**	80.168	(4.517, 1422.9)	**0.003**
**Male sex**	1.710	(0.812, 3.601)	0.157
**Age at 3rd dose [years]**	0.975	(0.948, 1.002)	0.074
**Body mass index [kg/m^2]**	0.991	(0.928, 1.058)	0.783
**eGFR at vaccination [ml/min/1.73m^2]**	1.021	(1.005, 1.036)	**0.009**
**Baseline anti-HLA antibodies positivity**	2.104	(1.019, 4.343)	**0.044**
**Timepoint – 3 months**	6.261	(4.649, 8.433)	**<0.001**
**Timepoint – 6 months**	8.092	(5.905, 11.089)	**<0.001**
**Timepoint – 12 months**	10.793	(7.265, 16.034)	**<0.001**
**Reduced dose of MMF/MPA**	0.310	(0.076, 1.255)	0.100
**Full dose of MMF/MPA**	0.269	(0.081, 0.889)	**0.032**
**Second booster dose (0 – 2 months)**	2.078	(0.730, 5.912)	0.170
**Second booster dose (2 – 4 months)**	2.712	(1.596, 4.611)	**<0.001**
**COVID-19 infection (0 – 3 months)**	8.519	(4.798, 15.125)	**<0.001**
**COVID-19 infection (3 – 6 months)**	5.623	(2.641, 11.972)	**<0.001**
**COVID-19 infection (6 – 9 months)**	6.601	(3.107, 14.024)	**<0.001**
**COVID-19 infection (9 – 12 months)**	5.740	(2.414, 13.646)	**<0.001**
**COVID-19 infection (12 – 15 months)**	3.328	(1.047, 10.577)	**0.042**
**COVID-19 infection (more than 15 months)**	2.265	(0.731, 7.021)	0.156

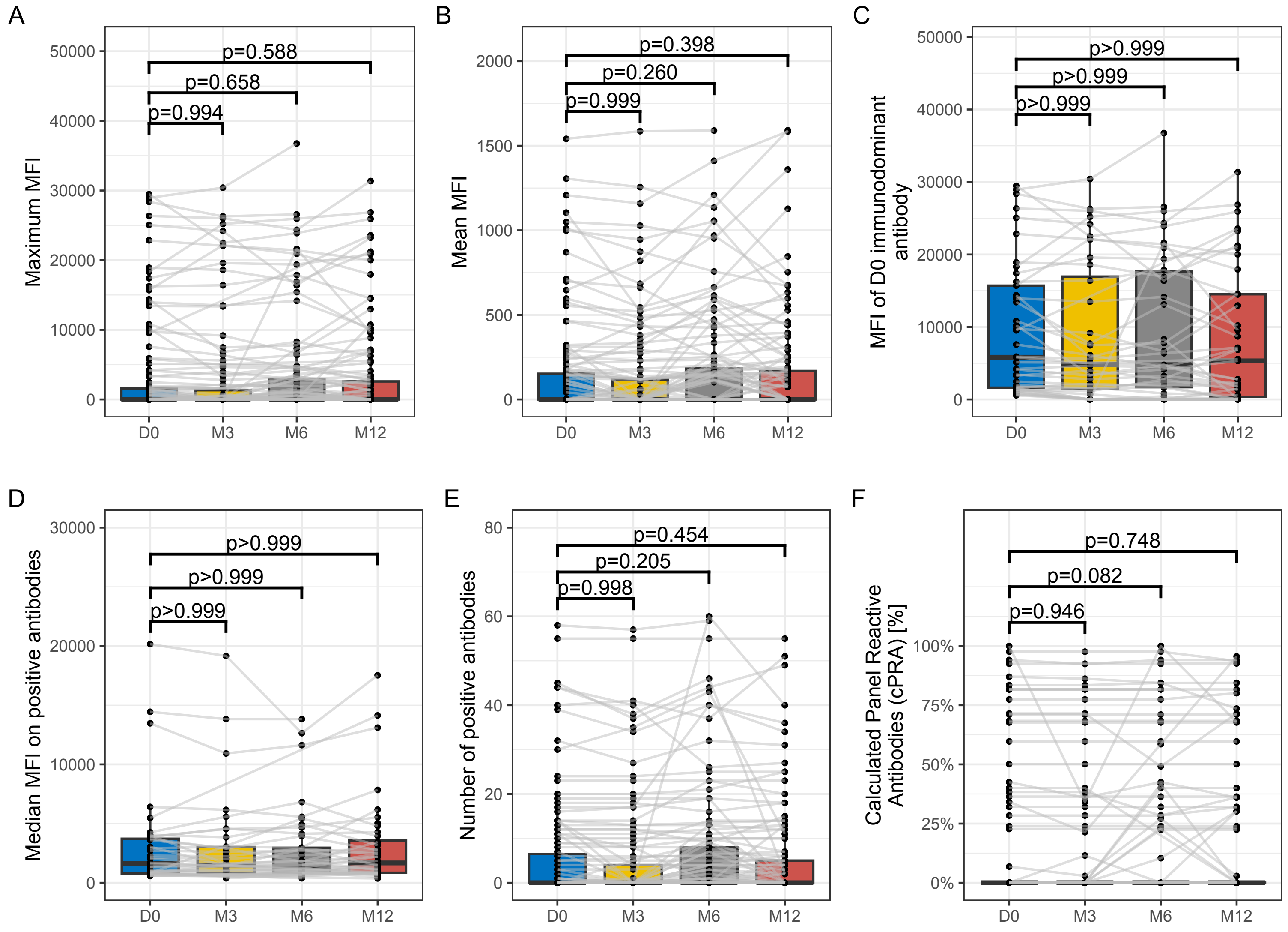

Figure S1. A sensitivity analysis of anti-HLA antibody dynamics during the follow-up when an alternative cut-off (MFI > 500) is used to define antibody positivity. X-axis of all panels shows 4 timepoints of the study: D0 – baseline, M3 – 3-months visit, M6 – 6-months visit, and M12 – 12months visit. The p-values show one-sided Wilcoxon tests comparing baseline and one of three timepoints. (A) Peak MFI, (B) Mean MFI, (C) MFI of immunodominant antibody at baseline, (D) Median MFI of positive antibodies, (E) Counts of positive antibodies, (F) Calculated Panel Reactive antibodies (cPRA).
